# Whole Exome Sequencing Identifies a Novel Predisposing Gene, MAPKAP1, for Familial Mixed Mood Disorder

**DOI:** 10.3389/fgene.2019.00074

**Published:** 2019-02-15

**Authors:** Chunxia Yang, Suping Li, Jack X. Ma, Yi Li, Aixia Zhang, Ning Sun, Yanfang Wang, Yong Xu, Kerang Zhang

**Affiliations:** ^1^Department of Psychiatry, First Hospital of Shanxi Medical University, Taiyuan, China; ^2^McGovern Medical School, University of Texas Health Science Center at Houston, Houston, TX, United States; ^3^School of Statistics, Shanxi University of Finance and Economics, Taiyuan, China; ^4^Nuring College of Shanxi Medical University, Taiyuan, China

**Keywords:** mood disorder, pedigree-based analysis, whole exome sequencing, rare variants, validation

## Abstract

**Background:** Mood disorder is ranked seventh among the worldwide causes of non-fatal disease burden and is generally believed to be a heritable disease. However, there is still a substantial portion of the heritability yet to be discovered, despite the success of genome-wide association studies (GWAS) for mood disorder. A proportion of the missing heritability may be accounted for by rare coding variants segregating in families enriched with mood disorder.

**Methods:** To identify novel variants segregating with mood disorder, we performed whole-exome sequencing on genomic DNA for a multigenerational family with nine members affected with mood disorder. We prioritized potential causal variants within the family based on segregation with mood disorder, predicted functional effects, and prevalence in human populations. In addition, for the top-ranked candidate variant, we conducted validation *in vivo* to explore the pathogenesis of mood disorder.

**Results:** We identified and ranked 26 candidate variants based on their segregation pattern and functional annotations. The top-ranked variant, rs78809014, is located in intron 7 of the MAPKAP1 gene. The expression levels of MAPKAP1 in peripheral blood of both major depression disorder (MDD) patients and depressive-like mice ventral dentate gyrus were significantly higher than that in the corresponding controls. In addition, the expression level of MAPKAP1 were correlated with antidepressant response.

**Conclusions:** Although the exact mechanisms in the family remain to be elucidated, our data strongly indicate a probable role of the variant, rs78809014, in the regulatory process of the expression of MAPKAP1 and thus in the development of mood disorder in familial mood disorder.

## Introduction

Mood disorders are a kind of serious mental illness and are characterized by high incidence, recurrence, and suicide rate (Ogasawara et al., 2018). Among different types of mood disorders, bipolar disorder (BD) affect ~1% of the population, causing severe psychosocial disturbance and requiring life-long treatment (Kanba et al., 2013; Stahl et al., 2013). On the other hand, major depression disorder (MDD) is a common disease with lifetime prevalence of around 10% (Motomura and Kanba, 2013). The social and financial burden due to these disorders is large and current treatments are still insufficient. Although the etiology of mood disorders remains largely unknown, a genetic component has been strongly suggested by family and twin studies. The heritability of mood disorders ranges from ~37% (95% Cl 31–42) for major depressive (Flint and Kendler, 2014), to 75% for bipolar disorder (Sullivan et al., 2012). In the last decade, many single nucleotide polymorphisms (SNP) and *de novo* or inherited copy number variations (CNV) have been found to be associated with mood disorders by genome-wide association studies (GWAS) and CNV analysis using DNA microarray (Kato, 2015). Despite of the success of GWAS, the identified SNPs and CNVs reaching a genome-wide significance level that are validated by independent studies so far can explain only a small portion of the heritability (Peterson et al., 2017; Xiao et al., 2017). It is generally believed that the degree of genetic heterogeneity is remarkably higher than previously thought for most of mood disorders, and the overall genetic structure probably has a polygenic component that contributes only a small portion of the overall liability. The rest of variance that cannot be explained by variants identified by GWAS, known as “missing heritability” may be accounted for by loci with modest to large effects (Collins et al., 2013; Cruceanu et al., 2013). Because GWASs focus on common variants, it is believed that low frequency (0.5~5%) and rare (<0.5%) variants could explain the missing heritability. Rare variants are known to play an important role in many Mendelian disorders and rare forms of common disease with high penetrance (Keinan and Clark, 2012; Zuk et al., 2014). Recent empirical evidence also shows that low-frequency and rare variants are associated with complex diseases (Goes et al., 2016).

With the advance of second-generation of DNA sequencing technologies, detection of rare variants has become increasingly feasible. Because rare variants have an extremely low frequency in general populations, one of the ideal study designs for detecting rare variants is to utilize pedigrees with a significant number of affected individuals (Roach et al., 2010). Availability of second-generation whole genome sequencing (WGS) or whole exome sequencing (WES) now permits the study of rare SNVs and small insertions/deletions (in/dels) in a systematic genome-wide manner (Roach et al., 2010; Ament et al., 2015). Studies using WGS or WES have been conducted for adult BD to search for highly penetrant rare variants (in 1% of population) with some success (Kato, 2015; Zhang et al., 2018). Collins et al. (2013) genotyped 46 individuals in a three-generation Old Order Amish pedigree with 19 affected (16 BP and 3 MD) and 27 unaffected subjects, and suggested that family based studies of the combined effect of common and rare CNVs at many loci may represent a useful approach in the genetic analysis of disease susceptibility of mental disorders. Although WGS has many advantages, such as allowing examination of both coding and non-coding regions (e.g., regulatory regions), WES is more cost effective, has much less computational burdens, and can quickly and effectively identify common and rare coding variants. In addition, in a large scale study of BD using WGS of 200 individuals from 41 families with BD, it was shown that an excess of rare variants in pathways associated with γ-aminobutyric acid and calcium channel signaling (Ament et al., 2015). In a recent study, Goes et al. (2016) performed exome sequencing of 36 affected members with BD from eight multiplex families, tested rare, segregating variants in three independent case-control samples consisting of 3,541 BD cases and 4,774 controls, and found 84 rare (frequency <1%), segregating variants that were bioinformatically predicted to be damaging (Goes et al., 2016).

In this study, we recruited a mood disorder-affected Chinese pedigree and sequenced the exomes of 22 subjects in this pedigree, which include 9 mood disorders and 13 unaffected members to explore novel genetic alterations predisposing individuals to the familial mood disorder. We also conducted validation *in vivo* from the perspective of genetics, to explore the pathogenesis of mood disorder.

## Materials and Methods

### Subjects

**Figure 1:**
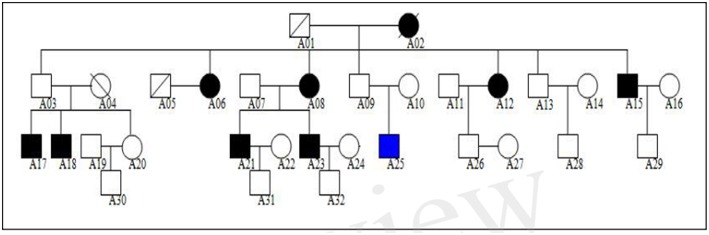
Northern Chinese family of ethnic Han origin in which 9 individuals (5 males and 4 females) affected with MDD or BD. The proband of this pedigree was A21, who was diagnosed with BD. None of these individuals displayed atypical findings on neurological examination, but one of them (A25) had mild intellectual disability since childhood.

We studied a Northern Chinese family of ethnic Han origin in which 9 individuals (5 males and 4 females) affected with MDD or BD (Figure 1). We recruited this family through a proband (A21), who was diagnosed with BD at the age of 22. Clinical diagnosis was made between March 2013 and February 2015 by at least two consultant psychiatrists according to Diagnostic and Statistical Manual of Mental Disorders Fourth Edition (DSM-IV) criteria for MDD (American Psychiatric Association, 2000). The affected individuals were also assessed with the Chinese Version of the Modified Structured Clinical Interview for DSM-IV TR Axis I Disorders Patient Edition (SCID-I/P, 11/2002 revision).All affected individuals had no other diseases, except for two of them who were with hypertension but in a stable condition. The 13 unaffected members of the family had no mental illness or other diseases.

In order to explore the functional impacts of the candidate gene identified from the pedigree, we utilized the data from one of our previous studies with 30 MDD. Details on the collection and diagnosis of the subjects were given in Sun et al. (2016). In short, all the patients were assessed by well-trained research assistants with background in psychology or psychiatry using the 17-item Hamilton depressive scale (HAMD-17) before and after a 8-week antidepressant treatment (SSRIs).

Among the 30 MDD patients, initial doses increased to curative doses administered for the next 2–4 weeks. The specific doses (maintenance doses and increased doses) were adjusted according to side effects and clinical assessment. If necessary, small doses of benzodiazepines were prescribed for agitation, but not persistent over 3 days. Twenty-two patients were remitted after 8-week antidepressant treatment (HAMD < 7). Five patients did not underwent the 8-week antidepressant and three did not meet the remitted individuals after the 8-week antidepressant treatment. Twenty-two healthy volunteers age and gender matched were selected controls were selected from 86 healthy volunteers. None of these controls had any family history of major psychiatric disorders (schizophrenia, bipolar disorder, MDD, and so on). All healthy controls did not have any history of blood transfusion or severe traumatic event within 1 month.

The study was approved by the Medical Research Ethics Committee of Shanxi Medical University. All subjects gave written informed consent.

### Exome Sequencing Followed by Quality Control and Statistical Analyses

#### Exome Capture and Sequencing

Blood samples for 22 family members (affected: A06, A08, A12, A15, A17, A18, A21, A23, A25; unaffected: A03, A07, A09, A10, A11, A13, A14, A16, A20, A22, A28, A29, A30) were submitted to MyGenomics (Bejing, China) for Genome Analysis. DNA was extracted from (5) mL aliquots using the CTAB approach. Each DNA sample was run on a (1) % agarose gel to determine if it was of high quality by analyzing the degradation degree and whether it was contaminated by RNA. The purity of DNA was analyzed with Nanodrop. The qualified DNA samples should be >1.5 μg, had no degradation or RNA contamination, and had a 260/280 ratio between 1.8 and 2.0. The quantified genomic DNA was sheared to a fragment length between 180 and 280 base pairs using focused acoustic energy (Covaris). The samples were then end repaired, A-tailed and ligated with specific adapters and multiplexing indexes.

The amplified DNA was captured with a polling biotin labeled probe (up to 543,872) for liquid-phase hybridization. The probes were designed to tile along 20,965 genes containing 334,378 exons. The capture experiment was conducted according to manufacturer's protocol. In brief, 1 μg DNA library was mixed with Buffer BL and GenCapgenepanel probe (MyGenostics, Beijing, China), and heated at 95°C for 7 min and 65°C for 2 min on a PCR machine; 23 μl of the 65°Cprewarmed Buffer HY (MyGenostics, Beijing, China) was then added to the mix, and the mixture was held at 65°C with PCR lid heat on for 22 h for hybridization. Fifty microliters MyOne beads (Life Technology) was washed in 500 μl 1X binding buffer for three times and resuspended in 80 μl 1X binding buffer. Sixty-four microliters 2X binding buffer was added to the hybrid mix, and transferred to the tube with 80 μl MyOne beads. The mix was rotated for 1 h on a rotator. The beads were then washed with WB1 buffer at room temperature for 15 min once and WB3 buffer at 65°C for 15 min three times. The bound DNA was then eluted with Buffer Elute. The eluted DNA was finally amplified for 15 cycles using the following program: 98°C for 30 s (1 cycle); 98°C for 25 s, 65°C for 30 s, 72°C for 30 s (15 cycles); 72°C for 5 min (1 cycle). The PCR product was purified using SPRI beads (Beckman Coulter) according to manufacturer's protocol. The enrichment libraries were sequenced on Illumina HiSeq 2000 sequencer for paired read 100 bp.

#### Quality Control, Variants Calling, and Annotation

High-quality reads were retrieved from raw reads by filtering out the low quality reads and adaptor sequences using the Solexa QA package and the cutadapt program, respectively. Reads were first aligned using the Burrows-Wheeler Algorithm (BWA) (http://bio-bwa.sourceforge.net/) to the human reference genome (GRCh37). The GATK (http://www.broadinstitute.org/gsa/wiki/index.php/Home_Page) package was then used to align the clean read sequences to the human reference genome (GRCh37). The identified SNPs and InDels were then filtered out if: (a) mapping qualities < 30; (b) the Total Mapping Quality Zero Reads < 4; (c) approximate read depth < 5; (d) QUAL < 50.0; or (e) phred-scaled *p*-value using Fisher's exact test to detect strand bias >10.0. The variants were then annotated using the ANNOVAR (http://annovar.openbioinformatics.org/en/latest/) with multiple databases, including 1000genome (http://www.1000genomes.org/), dbSNP (http://www.ncbi.nlm.nih.gov/projects/SNP/), EXAC (http://exac.broadinstitute.org/), Inhouse (MyGenostics), HGMD (http://www.biobase-international.com/product/hgmd). Non-synonymous variants were evaluated using four algorithms Ployphen SIFT (http://sift.jcvi.org/), PolyPhen-2 (http://genetics.bwh.harvard.edu/pph2/), and MutationTaster (http://www.mutationtaster.org/).

#### Filtering Based on Coverage, Functions, and Prevalence

Given our interest in identifying functional, rare or even family-specific variants, we applied a second process of filtering by removing those that were synonymous, with allele frequency > 5% in any of the mutation database, or with depth of coverage <6 or mutation ratio <30%.

#### Filtering Based on Segregation Pattern and Genetic Model

The segregation pattern of the MDD/BD in the family suggested a high, but not fully-penetrant dominant model because the father (A03) of the two affected sons (A17 and A18) were not affected. Also, we assumed that the causal variant segregated within the family members but not the marry-ins. Therefore, we first identified all invariants that were shared by all affected members and the father (A03) of A17 and A18; then we excluded variants that appeared in the five marry-ins.

#### Prioritizing and Ranking Using Mendelscan

Following the strategy proposed in the program package MendelScan (Koboldt et al., 2014), we prioritized potential causal variants within the family based on segregation with mood disorder, predicted functional effects, and prevalence in human populations. The segregation score, population score, and the annotation score of the candidate variants were calculated using the MendelScan approach.

### Differential Expression Analysis

The Kolmogorov-Smirnov test was used to test the normality of the expression data. Differential expression analysis for MDD vs. control groups and treated vs. untreated MDD were performed using two-sample *t*-test. All analyses were conducted with SPSS (version 17.0) and GraphPad Prism (version 5.0). Data were expressed as the means ± standard deviations (SD) unless otherwise indicated. The demographic data of the two groups were analyzed using *t*-test and ChiSquare Test. *P* < 0.05 were considered statistically significant.

### RNA Extraction

Blood samples of 22 MDD patients before and after a 8-week treatment and 22 healthy controls were collected using EDTA anticoagulant tube and processed within 3 h. Peripheral blood leukocytes were isolated by centrifugation from the fresh blood sample, and stored at −80°C in fresh RNase/DNase-free 2 ml microcentrifuge tube. Total RNAs were extracted from the peripheral blood leukocytes with the TRIzol (Invitrogen; USA) with on-column DNase I treatment according to the manufacturer's protocol. The integrity of total RNA was evaluated by denaturing agarose gel electrophoresis. RNA was further purified using an RNeasy mini kit (Qiagen, Valencia, CA, USA) according to the manufacturer's instructions.

### qPCR and Comparison of Gene Expression

The expression of MAPKAP1 gene of 22 MDD patients and 22 health controls was analyzed by real-time quantitative PCR (qRT-PCR). cDNA was synthesized using a High Capacity RNA-to-cDNA Kit (Invitrogen; USA) as described by the manufacturer. The primers used for MAPKAP1 are listed in Table 1. PCR was performed using a 7900HT real-time PCR machine (Applied Biosystems; USA) for 2 min at 50°C, 2 min at 95°C, and then 40 cycles consisting of 15 s at 95°C, 60 s at 60°C, followed by a subsequent standard dissociation protocol to ensure that each amplicon was a single product. All quantifications were normalized to GAPDH. The qRT-PCR was performed in triplicate for each of the three independent samples. The expression of MAPKAP1 was measured using the miScript system(QIAGEN, CA) (including miScript Reverse Transcription kit, miScript Primer assays and miScript SYBR Green PCRkit) as described by the protocol provided by the company. Small nuclear RNA U6 was used for normalization. The threshold cycle (CT) was defined as the fractional cycle number at which the fluorescence passes the fixed threshold. The comparative Ct (2∧–ΔΔCT) method was used for quantification of transcripts.

**Table 1:** Sequences for primers of MAPKAP1.

**Primer for qRT-PCR**	**Primer sequences**
MAPKAP1	5′ - ACTCATCTCCTGGTCTGACATC−3′
	5′ - TCTTCGCTTCACTGCCTTC−3′

### GEO Data Analysis

We also analyzed the expression data of MAPKAP1 from GEO in ventral dentate gyrus from adults mice subjected to chronic corticosterone (CORT) to induce depression-like behaviors, followed by selective serotonin reuptake inhibitor fluoxetine (Samuels et al., 2014). All mice were 7–8 weeks old and weighed 23–35 g at the beginning of the treatment. Fifteen mice were treated with chronic corticosterone (CORT), which induced depression-like behaviors with increased immobility in the Forced Swim Test (FST) and anxiety-like behavior with increased latency to eat in the Novelty Suppressed Feeding Test (NSF). These features can then be reversed with fluoxetine (FLX) for 21 days. However, a minority of mice (4 out of 15) appeared to respond to FLX in the NSF but not in the FST, which were not used for microarray experiments, such that 11 (7 appeared to respond to FLX, 4 appeared to resist to FLX) out 15 fluoxetine treated mice were used for microarrays. Eight randomly chosen mice not treated with FLX were used as representative controls for microarray studies. Student's *t*-test was performed to compare the expression levels between MDD mice group and control group, and between Responder group and resistant group, control group.

## Results

The genogram of the pedigree is given in Figure 1. Blood samples were available for 9 affected and 13 unaffected members (A03, A07, A09, A10, A11, A13, A14, A16, A20, A22, A28, A29, A30) of the family, and we performed WES on these family members (Figure 1).

The clinical characteristics of the affected family members are provided in Table 1. None of these individuals displayed atypical findings on neurological examination, except that one of them (A25) had mild intellectual disability since childhood. Two of the affected members had hypertension. There were nine affected family members, seven of them diagnosed major depression, and one diagnosed bipolar disorder (Table 2).

**Table 2:** Clinical characteristics of the affected members of the pedigree.

**Patient**	**Age at onset, yr**	**Past medical history**	**Mood disorder**	**History of treatment**
A06	>25	Hypertension	MD	N
A08	>25	–	MD	Y
A12	>25	–	MD	N
A15	< 25	Hypertension	MD	N
A17	< 25	–	MD	N
A18	< 25	–	MD	Y
A21	< 25	–	BD	Y
A23	< 25	–	MD	Y
A25	< 25	–	MD	N

### QC of Sequencing Data

We attained relatively high coverage of the exome, with each sample achieving, in average, 12,638 Mbp. On average, 94.6 and 89.0% base pairs had Phred values greater than 20 and 30, respectively. On average, 99.8% of the base pairs passed the strict quality control filters for each sample. On average, about 84% of base pairs were mapped to the reference genome, 34% to the targeted region, the exomes, and the average depth was 69.6 and covered 98.5% of the exomes. On average, 96.1, 91.9, and 84.3% of the exomes had sequencing depth 4X, 10X, and 20X, respectively. On average, 84.37% of reads were successfully aligned, 10.74% PCR replicates per sample, resulting in 98.52% coverage, with 84% of the exome being covered by 20 or more reads. The detailed quality control metrics were given in Table 3.

**Table 3:** Average variants coverage by sample.

**Sample**	**Coverage**	**Coverage_passed QC**	**Coverage_passed filter**
A12	75.0	76.8	76.9
A13	78.9	80.6	78.9
A28	77.7	79.5	78.1
A30	71.1	72.9	70.6
A14	69.3	71.4	71.8
A03	65.5	67.4	68.0
A06	67.2	69.3	70.9
A08	56.1	58.2	60.2
A15	46.4	48.6	52.8
A16	78.7	80.7	81.1
A18	58.5	60.8	65.4
A21	58.8	60.6	62.9
A07	57.5	59.4	62.1
A17	77.3	79.4	77.2
A09	65.0	66.8	66.1
A23	54.2	56.3	58.3
A10	72.6	74.2	74.7
A20	69.6	71.6	69.8
A29	64.8	66.8	67.3
A11	79.6	81.5	80.6
A22	71.0	73.0	73.4
A25	69.4	71.5	72.0

### Variants Calling and QC

There were 115,915 variants (102,717 SNVs, and 13,198 InDels) called that were variable in at least one sample. For those called variants, the average coverage per sample was between 46X and 79X. Of those variants, the majority were within exons (49%), and introns (30%). About 8% of those variants were in regions not considered to be within or around known genes (Table 4). After applying stringent quality control filters, 101,126 variants remained, including 93,071 SNVs, and 8,055 InDels. For those high quality variants, the average coverage was between 48X and 81X. The majority of those variants were within exons (51%) and introns (31%), while 6.5% within intergenic regions (Table 4).

**Table 4:** Distribution of variant types.

**Variant-type**	**All-called-variants**	**Variants-passed-QC-filters**	**Variants-passed-functional-filter**
Intronic	34,660 (30.20)	30,969 (30.62)	7,225 (33.91)
Exonic	55,814 (48.63)	51,666 (51.09)	8,071 (37.88)
ncRNA_exonic	3,644 (3.18)	2,672 (2.64)	798 (3.75)
Splicing	2,392 (2.08)	2,010 (1.99)	534 (2.51)
ncRNA_intronic	3,032 (2.64)	2,249 (2.22)	796 (3.74)
UTR3	2,667 (2.32)	2,338 (2.31)	501 (2.35)
UTR5_UTR3	7 (0.01)	6 (0.01)	0
Upstream	582 (0.51)	448 (0.44)	128 (0.60)
UTR5	2,075 (1.81)	1,788 (1.77)	412 (1.93)
ncRNA_splicing	94 (0.08)	67 (0.07)	28 (0.13)
Upstream_downstream	29 (0.03)	22 (0.02)	7 (0.03)
Intergenic	9,315 (8.12)	6,584 (6.51)	2,686 (12.61)
Exonic_splicing	81 (0.07)	73 (0.07)	15 (0.07)
Downstream	371 (0.32)	234 (0.23)	105 (0.49)
Total	114,763	101,126	21,306

### Filtering Based on Coverage, Functions, and Prevalence

This filtering process yielded 21,306 variants, including 18,464 SNVs, and 2,842 InDels, with the average coverage between 53X and 81X. Among those variants, 8,071 (38%) were within exons, 7,225 (34%) within introns, and 2,686 (13%) within intergenic regions (Table 4).

### Filtering Based on Segregation Pattern and Genetic Model

We assumed that the causal variant segregated within the family members but not the marry-ins (see Methods for details). We first identified 475 variants that were shared by all affected members and the father, A03, of A17 and A18; then we excluded those variants among 1,104 variants that were found in all the five marry-ins, yielding a final set of 26 candidate variants.

### Prioritizing and Ranking Candidate Variants

To prioritize those 26 candidate variants, we calculated the segregation score, population score, and the annotation score of them using the MendelScan approach (Koboldt et al., 2014). The results are summarized in Table 5. The top-ranked variants is rs78809014 (chr9; 128305252;128305252; C;G) with population-score is 0.02, and located at the seventh intron of gene MAPKAP1. The gene is a key component in mTOR signaling pathway, which has great potential for the identification of new therapeutic targets for the development of antidepressant drugs in MDD and in response to antidepressants (Szewczyk et al., 2015; Liu et al., 2016). Specifically, the top variant (rs78809014) had a perfect association with the affection status: all affected had the mutation and all unaffected except A03 had the normal allele.

**Table 5:** Scores of the family separation analysis.

**No**	**Loci**	**gene**	**Overall_score**	**ann_score**	**popu_score**	**seg_score_dom**	**seg_score**	**Gene-region_effect**	**Snp138Non-flagged**	**Mutation_type**	**1000g2012apr**
1	chr9;128305252;128305252; C;G	MAPKAP1	0.0002	0.01	0.02	1	0.001953	intronic_	rs78809014	SNV	0.008786
2	chr21;14982716;14982716; T;C	POTED	4.80E-07	0.8	0.6	1.00E-06	0.001953	exonic _non-synony	rs201206142	SNV	–
3	chr5;115346245;115346245; T;G	LVRN	3.84E-08	1	0.6	6.40E-08	0.001953	intronic_-intron variant, missense	rs17138646	SNV	–
4	chr16;33629700;33629700; G;A	–	6.00E-09	0.01	0.6	1.00E-06	0.001953	intergenic_-	rs2019670	SNV	–
5	chr14;77579754;77579754; T;C	CIPC	1.02E-09	0.01	0.02	5.12E-06	0.007813	intronic_-	rs74069037	SNV	0.023962
6	chr22;22730853;22730853; G;GCC	–	1.00E-09	0.01	1	1.00E-07	0.001953	intergenic_-	–	indel	–
7	chr22;22730849;22730849; GTA;G	–	1.00E-09	0.01	1	1.00E-07	0.001953	intergenic_-	–	indel	–
8	chr22;22730891;22730891; G;C	–	6.00E-10	0.01	0.6	1.00E-07	0.001953	intergenic_-	rs201626791	SNV	–
9	chr22;22730869;22730869; G;A	–	6.00E-10	0.01	0.6	1.00E-07	0.001953	intergenic_-	rs114666913	SNV	–
10	chr22;22730860;22730860; C;T	–	6.00E-10	0.01	0.6	1.00E-07	0.001953	intergenic_-	rs117244515	SNV	–
11	chr16;33630022;33630022; A;T	–	6.00E-10	0.01	0.6	1.00E-07	0.001953	intergenic_-	rs2075113	SNV	–
12	chr16;33534209;33534209; T;A	LOC102724207	6.00E-10	0.01	0.6	1.00E-07	0.001953	ncRNA_intronic_-	rs111304289	SNV	–
13	chr2;170633023;170633023; T;TA	–	3.84E-10	0.01	0.6	6.40E-08	0.001953	intergenic_-	rs11422611	indel	–
14	chr22;22730829;22730829; G;A	–	2.00E-10	0.01	0.2	1.00E-07	0.001953	intergenic_-	rs140157920	SNV	0.000399
15	chr16;33402061;33402061; G;A	–	2.00E-10	0.01	0.02	1.00E-06	0.001953	intergenic_-	rs8047387	SNV	–
16	chr16;33534205;33534205; T;C	LOC102724207	6.00E-11	0.01	0.6	1.00E-08	0.001953	ncRNA_intronic_-	rs75274563	SNV	–
17	chr21;28215826;28215826; C;CACA	ADAMTS1	3.07E-11	0.01	0.6	5.12E-09	0.007813	intronic_-	rs373460567	indel	–
18	chr2;136530157;136530157; G;A	UBXN4	1.28E-11	0.01	0.02	6.40E-08	0.003906	intronic_-	rs78878675	SNV	0.039736
19	chr2;136511886;136511886; G;A	UBXN4	1.28E-11	0.01	0.02	6.40E-08	0.003906	intronic_-	rs74265494	SNV	0.039736
20	chr16;71100830;71100830; T;C	HYDIN	6.00E-12	0.01	0.6	1.00E-09	0.001953	intronic_-	rs146519470	SNV	–
21	chr2;227966298;227966298; T;TA	COL4A4	5.24E-12	0.01	0.2	2.62E-09	0.03125	intronic_-	rs59443812	indel	–
22	chr19;33490480;33490480; G;A	RHPN2	2.00E-12	0.01	0.2	1.00E-09	0.001953	intronic_-	rs74603434	SNV	-
23	chr14;106994063;106994063; C;T	–	4.80E-13	0.01	0.6	8.00E-11	0.000977	intergenic_-	rs4774148	SNV	–
24	chr3;129108158;129108158; A;AG	RPL32P3	1.57E-14	0.01	0.6	2.62E-12	0.015625	ncRNA_intronic_-	rs58915955	indel	-
25	chr17;12859125;12859125; C;CG	ARHGAP44	1.01E-16	0.01	0.6	1.68E-14	0.03125	intronic_-	rs3214256	indel	-
26	chr21;45649494;45649494; A;G	ICOSLG	8.05E-21	0.01	0.6	1.34E-18	0.003906	intronic_-	rs7278772	SNV	–

### Clinical Characteristics of the MDD Cases and Controls

As shown in Table 6, 22 MDD patients underwent a 8-week antidepressant treatment (sole SSRI). All the patients and controls were of Han nationality, and there were no statistically significant differences in age, sex or residential locations between MDD patients and healthy controls. The first mean score of HAMD was 19.86 ± 2.51.

**Table 6:** Clinical characteristics of MDD patients and healthy controls.

**Variables**	**Cases (*n* = 22)**	**Controls (*n* = 22)**	***t/**χ^2^*****	***P***
Age, years, (SD)	43.05 ± 9.87	43.55 ± 10.44	0.164	0.871
Male	7	6	0.323	0.748
Female	15	16	–	–
Education, years, (SD)	11.14 ± 3.47	10.82 ± 3.57	−0.300	0.766

### Expression of MAPKAP1 in MDD and Control

**Figure 2:**
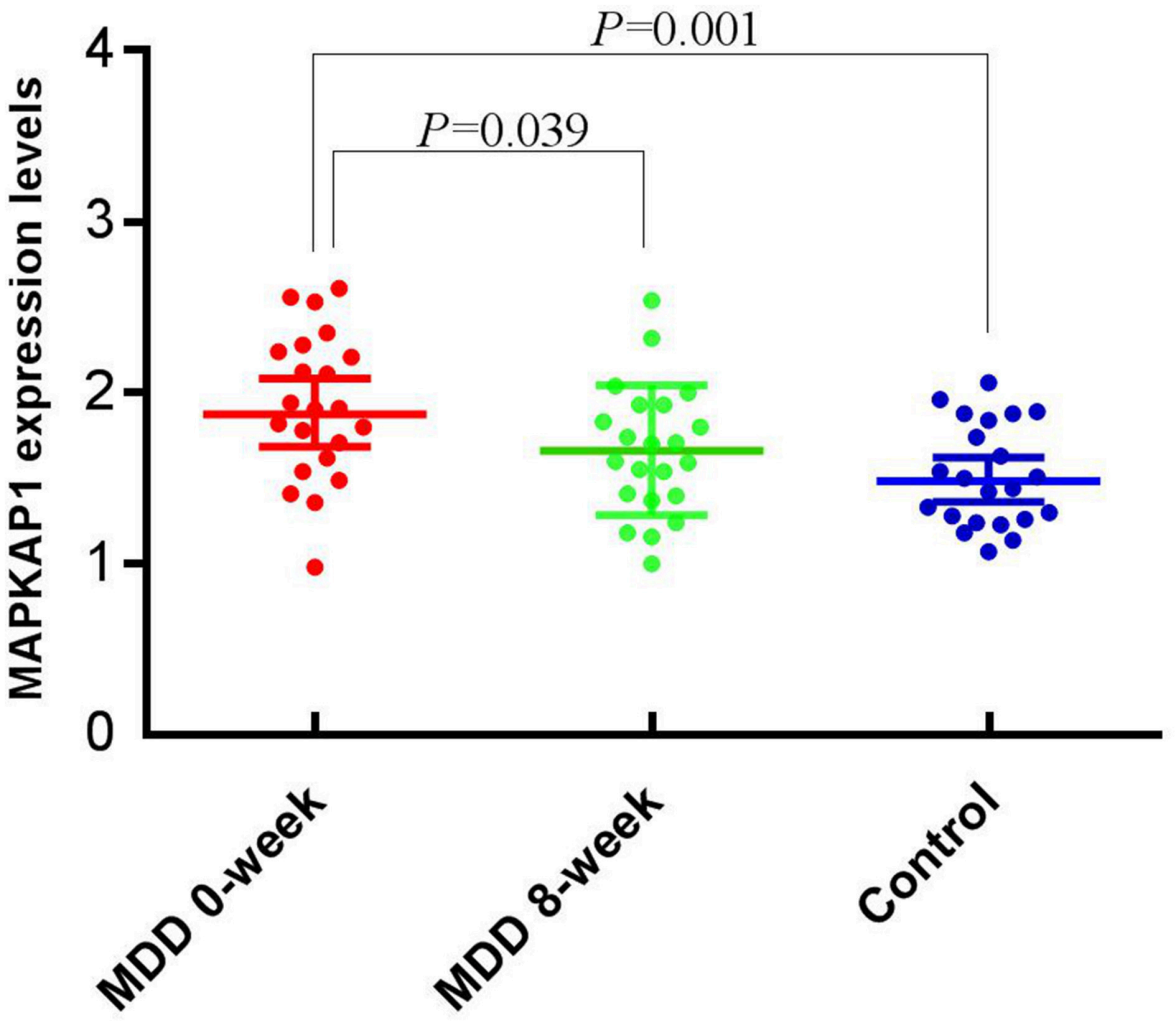
Expression levels of MAPKAP1 between before and after treatment, and between cases and controls.

All the expression data follow normal distribution (*P* > 0.05). The mean expression level of the MDD group before treatment (1.92 ± 0.42) was significantly higher than that of the control group (1.52 ± 0.30), (*t* = 3.652, *P* = 0.001); and was also significantly higher than that after treatment (1.66 ± 0.38), (*t* = 2.131, *P* = 0.039) (Figure 2).

### Expression of MAPKAP1 in MDD and Control Mice

**Figure 3:**
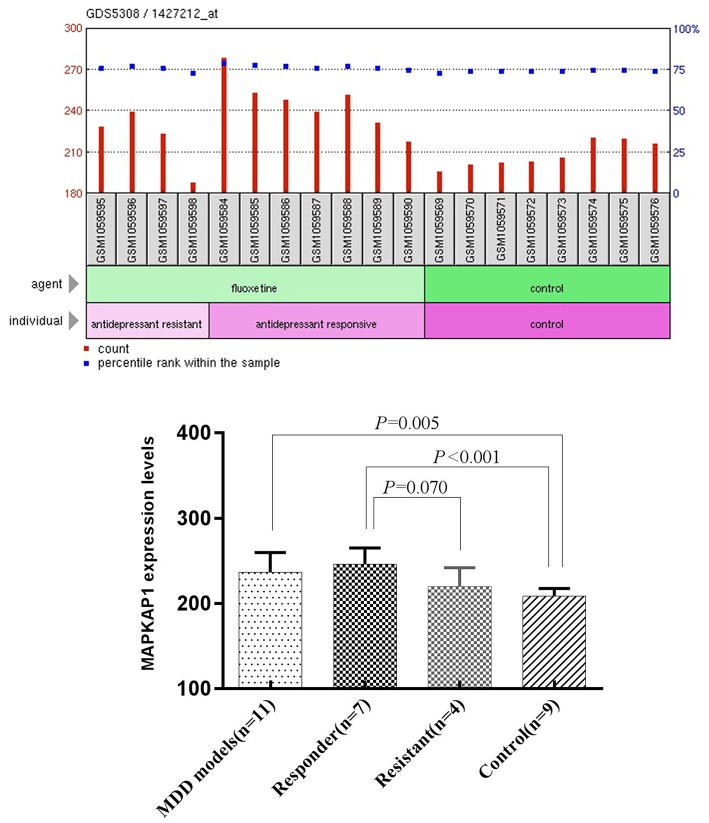
The expression levels of MAPKAP1 in ventral dentate gyrus of MDD mice models, Responders, resisitants and controls **(Left)** and *t*-test results **(Right)**.

We performed One-Sample Kolmogorov-Smirnov test for the expression data in each of the four groups separately and found that each of them follow a normal distribution (*P* > 0.05). The mean expression level of MAPKAP1 in ventral dentate gyrus of MDD mice (236.46 ± 23.17) was significantly higher than that of the control group (208.47 ± 9.23), (*t* = −3.216, *P* = 0.005). The mean expression level of MAPKAP1 in ventral dentate gyrus of Responder group (245.90 ± 19.07) was significantly higher than that of the control group (208.47 ± 9.23), (*t* = 4.946, *P* < 0.001) and was also marginally higher than that resisitant group (219.95 ± 22.14), (*t* = −2.055, *P* = 0.070) (Figure 3).

## Discussion

With only a small fraction of the predicted heritability being accounted for by variants identified through linkage analysis and GWAS, it is believed that mood disorder is highly genetically heterogeneous and its genetic susceptibility factors may involve rare variants (Gershon, 2000). The pedigree we recruited was enriched with affected individuals and thus provided us with a great opportunity to reduce the genetic heterogeneity and identify rare or even family-specific variants responsible to mood disorder (Rao et al., 2017). Exome-sequencing is increasingly utilized to identify rare and likely disease-causing mutations in many neuropsychiatric disorders (Binder, 2012). The segregation pattern of mood disorder in this pedigree strongly suggests a high-penetrant dominant model. Based on this model and the segregation score, annotated functional score, and population score, we identified and ranked 26 candidate rare variants. These rare mutations were shared by affected family members and were absent in the unaffected family members (except for A03), which is in consistent with the currently favored hypothesis of oligogenic disease causation in BD (Gershon, 2000; Rao et al., 2017).

The top-ranked variant, rs78809014, is located in the intronic region of the MAPKAP1 gene. This variant may be regarded as being segregated perfectly with mood disorder in this pedigree, if it is deemed as not penetrant in A03 who was the father of two affected sons. rs78809014 may be regarded as a rare variant with a global MAF 0.0088 in the major populations. However, in the HapMap population CHB (Han Chinese in Beijing) the MAF is as high as 0.044 (https://www.ncbi.nlm.nih.gov/snp/?term=rs78809014). Indeed, to our best knowledge, none of the GWAS studies have identified rs78809014 as a susceptibility locus associated with the risk of mood disorders. We anticipate that the reasons why this not-so-rare variant (especially in the Chinese population) may have escaped from the genome-wide scans may be that mood disorders are complex diseases caused by a combinations of inherited variations, which often acting together with some environmental and/or behavioral factors (Li et al., 2010). It is possible that there was an additional genetic mutation shared by the affected individuals in the pedigree under study, which might be identified should we conducted a WGS. This additional mutation might act together with rs78809014 to cause the prevalence of mood disorder in this pedigree. It is also like that those affected may share a common environmental risk factor that interacts with rs78809014 in relation to the regulation of the expression of MAPKAP1.

MAPKA1 (also known as Sin1), is a key component of mTORC2 signaling complex which is necessary for AKT phosphorylation (Li et al., 2010; Machado-Vieira et al., 2015). Ketamine, which recently became one of most popular antidepressant medicines and has been proved to be effective, could rapidly activate the mammalian target of rapamycin (mTOR) pathway, leading to increased synaptic signaling proteins and increased number and function of new spine synapses in the prefrontal cortex of rats (Abelaira et al., 2014). Fluoxetine, the clinical commonly classic antidepressant medication, also has been proved to regulate mTOR signaling in a region-dependent manner in depression-like mice and mainly in the hippocampus (Liu et al., 2015). Some of us have previously reported that the key genes, AKT1 and GSK3B, of mTORC2 signaling appear to be associated with MDD in the Han Chinese population (Yang et al., 2010, 2012; Zhang et al., 2010; Liu et al., 2014). The characterization of the mTOR signaling pathway in depression and its action in response to antidepressants show great potential for the identification of new therapeutic targets for the development of antidepressant drugs (Szewczyk et al., 2015; Liu et al., 2016).

Although the variant rs78809014 is located in an intron and has not being annotated with any functional terms, we anticipate that it may have some kind of regulatory functional impact on the MAPKAP1 gene. Data from *in vivo* were supportive to our anticipation that the expression level of MAPKAP1 may be altered in the development of MDD. We compared the mRNA expression levels of MAPKAP1 in MDD patients vs. healthy controls and before and after treatment (8 weeks) for the MDD patients and found that MAPKAP1 was indeed overexpressed in MDD patients and treatment with (SSRI) could significantly reduce its expression level. Also, the expression levels of MAPKAP1 in responder mice ventral dentate gyrus were significantly higher than that in the control group, and was also marginally higher than that in resistant group. Recent studies show that antidepressant treatment such as imipramine inhibited PI3K/Akt/mTOR signaling (Jeon et al., 2011). Moreover, Lin et al. (2010) showed that sertraline exerts antiproliferative activity by targeting the mTOR signaling pathway in rat embryonic fibroblasts. Our data provided further evidence that the expression level of MAPKAP1 plays an important role in the pathology of MDD. In a future study, it will be highly desirable to investigate whether this variant affect the expression level of MAPKAP1 in the brain tissues and/or blood cells. Moreover, a more detailed expression analysis at the transcript level should be performed in future studies, because there exist 21 isoforms of the gene KAPMAP1. Specifically, in the qPCR experiments, more than five different transcripts were mapped and the results reflected an aggregative expression of those isoforms.

Not surprisingly, none of the candidate variants identified in this study was implicated in any of the 15 major GWASs on BD (Shinozaki and Potash, 2014). Because of the genetic heterogeneity of mood disorder, a single susceptibility variant may affect only a very small proportion in a population. Given the substantial evidence that the majority of causal genetic variants in BD are common with very small effect sizes (OR ¼ 1.05 to 1.20), individual rare variants can hardly reach a genome-wide significance level required in a typical GWAS. It is the advantage of a family-based WES or WGS study to identify rare, moderate to high penetrant variants susceptible to complex diseases such as mood disorders.

The pedigree we have studied included multiple affected individuals with BD or MDD. Although it has been believed that focusing on a single subtype of a disease to reduce the phenotypic heterogeneity will increase the chance of identifying genetic factors, increasing evidence has indicated that familial co-aggregation or comorbidity between these disorders is mainly attributable to overlapping genetic influences (Smoller and Finn, 2003; Chang et al., 2013). A recent large-scale GWAS across the five disorders, MDD, BD, SZ, ADHD, and autism, identified SNPs at four loci that accounted for some of the shared variation across the disorders at *p* < 5 ^*^10^8^ (Cross-Disorder Group of the Psychiatric Genomics Consortium, 2013). Moreover, the proband of this pedigree was diagnosed with BD at the age of 22, the cut-off age for defining early onset (Grigoroiu-Serbanescu et al., 2014). It is believed that early-onset BD is more heritable and more severe than BD occurring at older age (Schürhoff et al., 2000; Grigoroiu-Serbanescu et al., 2001; Somanath et al., 2002). The etiologic mechanisms for BD are not well-understood, but empirical data consistently suggest the polygenic character of BD with estimated heritability ranging from 80 to 85% (Barnett and Smoller, 2009). We therefore strongly believe that there existed a genetic variant segregating along with and responsible to the mood disorder in this pedigree.

One of the limitations of exome sequencing is that it does not identify the non-coding and structural variants that could be found by WGS. Also, none of the current exome capture reagents cover 100% of the coding region. Nevertheless, we believe that the candidate variants identified in the current study warrant validations via family-based and/or population based studies of large sample size. Furthermore, the proposed susceptibility genes may be validated functionally for their role in the brain and the impact of the identified mutations on protein structure and function and/or expression levels of the corresponding transcripts.

## Author Contributions

KZ: Study concepts and definition of intellectual content; KZ and YX: Study design; AZ: Literature research; YW and SL: Clinical studies; NS: Experimental studies; CY and YX: Data acquisition; CY and JM: Data analysis and statistical analysis; CY: Manuscript preparation; CY and YL: Manuscript editing; AZ, YL, KZ, and YX: Manuscript review. All authors have approved the final article.

### Conflict of Interest Statement

The authors declare that the research was conducted in the absence of any commercial or financial relationships that could be construed as a potential conflict of interest.
